# Intensity modulated or fractionated stereotactic reirradiation in patients with recurrent nasopharyngeal cancer

**DOI:** 10.1186/1748-717X-6-22

**Published:** 2011-03-01

**Authors:** Falk Roeder, Felix Zwicker, Ladan Saleh-Ebrahimi, Carmen Timke, Christian Thieke, Marc Bischof, Juergen Debus, Peter E Huber

**Affiliations:** 1 Clinical Cooperation Unit Radiation Oncology, German Cancer Research Center (DKFZ), Heidelberg, Germany; 2 Department of Radiation Oncology, University of Heidelberg, Heidelberg, Germany

## Abstract

**Purpose:**

To report our experience with intensity-modulated or stereotactic reirradiation in patients suffering from recurrent nasopharyngeal carcinoma

**Patients and Methods:**

The records of 17 patients with recurrent nasopharygeal carcinoma treated by intensity-modulated (n = 14) or stereotactic (n = 3) reirradiation in our institution were reviewed. Median age was 53 years and most patients (n = 14) were male. The majority of tumors showed undifferentiated histology (n = 14) and infiltration of intracranial structures (n = 12). Simultaneous systemic therapy was applied in 8 patients. Initial treatment covered the gross tumor volume with a median dose of 66 Gy (50-72 Gy) and the cervical nodal regions with a median dose of 56 Gy (50-60 Gy). Reirradiation was confined to the local relapse region with a median dose of 50.4 Gy (36-64Gy), resulting in a median cumulative dose of 112 Gy (91-134 Gy). The median time interval between initial and subsequent treatment was 52 months (6-132).

**Results:**

The median follow up for the entire cohort was 20 months and 31 months for survivors (10-84). Five patients (29%) developed isolated local recurrences and three patients (18%) suffered from isolated nodal recurrences. The actuarial 1- and 2-year rates of local/locoregional control were 76%/59% and 69%/52%, respectively. Six patients developed distant metastasis during the follow up period. The median actuarial overall survival for the entire cohort was 23 months, transferring into 1-, 2-, and 3-year overall survival rates of 82%, 44% and 37%. Univariate subset analyses showed significantly increased overall survival and local control for patients with less advanced rT stage, retreatment doses > 50 Gy, concurrent systemic treatment and complete response. Severe late toxicity (Grad III) attributable to reirradiation occurred in five patients (29%), particularly as hearing loss, alterations of taste/smell, cranial neuropathy, trismus and xerostomia.

**Conclusion:**

Reirradiation with intensity-modulated or stereotactic techniques in recurrent nasopharyngeal carcinoma is feasible and yields encouraging results in terms of local control and overall survival in patients with acceptable toxicity in patients with less advanced recurrences. However, the achievable outcome is limited in patients with involvement of intracranial structures, emphasising the need for close monitoring after primary therapy.

## Background

Radiotherapy with or without simultaneous chemotherapy according to stage is the standard of care for primary nasopharyngeal cancer resulting in excellent local control and overall survival rates [1-6]. However, local or locoregional failure still represents a major failure pattern, especially in advanced T stage [7]. Although several treatment options for local relapses exist, including surgery, chemotherapy and reirradiation with various techniques, treatment remains challenging due to close organs at risk with high impact on functional outcome. While surgery is the preferable treatment option for regional lymph node failure in patients treated primarily with combined chemoradiation [8], its use in local recurrences of the nasopharynx itself often needs demanding procedures. Surgery is especially challenging in locally advanced lesions because of the difficult exposure of this region, and flawed with a high risk of functional deficits. Chemotherapy alone is the treatment of choice in patients with metastastic disease, but has to be considered as a palliative treatment option only for patients with localized recurrence not amendable to local treatment options, at least as long term survivors have rarely been described [9]. Because nasopharyngeal cancer is known to be sensitive to radiation therapy, reirradiation has been used in various approaches to treat local recurrences [7]. Brachtherapy techniques have been described either as intracavitary mould techniques for recurrent lesions confined to the nasopharynx [10] or by the use of interstitial gold grain implants for more advanced lesions, resulting in good local control and overall survival rates [11], but are restricted to a small number of highly specialized and experienced centers. Stereotactic single dose radiotherapy, also known as radiosurgery, has also been described as a valid treatment option for small recurrent lesions [12] but could result in significant toxicity due to the unfavourable radiobiology of single dose treatments if used for advanced lesions. Because fractionated external beam radiotherapy should offer a more favourable radiobiology in terms of toxicity, it has been used for almost two decades for the treatment of recurrent nasopharyngeal cancer. Although achieving acceptable results in terms of local control, a significant number of late sequelae has been reported especially in the early literature using 3-dimensional (3D) or even 2-dimensional (2D) radiation treatment techniques due to the lack of conformality with adaequate sparing of adjacent organs at risk [13-15]. However, radiation therapy techniques have emerged consistently over time, and the introduction of fractionated stereotactic or intensity-modulated radiotherapy should theoretically result in a more favourable balance between target coverage and sparing of adjacent organs at risk especially in complex shaped advanced recurrent nasopharyngeal lesions [16]. To confirm this assumption in daily clinical routine, we report our experience using intensity-modulated radiotherapy in the treatment of previously irradiated localized recurrences of nasopharyngeal cancer.

## Patients and Methods

**Figure 1:**
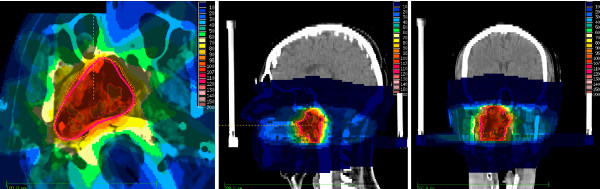
**Example for dose distribution in axial, sagittal and frontal view**. dotted line: 95% isodose.

A total of 17 patients with recurrent nasopharyngeal cancer have been treated in our institution with fractionated intensity modulated or stereotactic reirradiation. All patients suffered from histologically proven localized recurrent nasopharyngeal cancer without distant metastasis. Initial work-up included clinical examination, CT and/or MRI of the head and neck region, panendoscopy with histological confirmation, chest x ray or CT of the lung, abdominal ultrasound or CT and bone scan for the exclusion of distant metastases, laboratory examinations and review of the former radiotherapy reports. Median age at reirradiation was 53 years (range 23-67 years) and most patients (n = 14) were male. The majority of tumors showed undifferentiated histology (Grade III according to WHO classification, n = 14) and infiltration of cranial structures (rT4, n = 12). For detailed patient characteristics see table 1. Initial radiation treatment covered the primary tumor with a median dose of 66 Gy (50-72 Gy) and except in one patient, the bilateral cervical and supraclavicular nodal regions with a median dose of 56 Gy (50-60 Gy). The median time interval between the initial and present treatment was 52 months (6-132 months). Reirradiation was performed using step-and-shoot intensity modulated radiotherapy (IMRT) in 14 patients and fractionated stereotactic radiotherapy (FSRT) in 3 patients. The techniques of IMRT and FSRT used in our institution have been previously described [17-23]. Briefly, all patients were fixed in an individually manufactured precision head mask made of Scotch cast^® ^(3 M, St.Paul, Minneapolis, MN). With this immobilization system attached to the stereotactic base frame, contrast-enhanced CT- and MRI-images were performed with a slice thickness of 3 mm. After stereotactic image fusion based on the localizer-derived coordinate system, all critical structures as well as the target volumes were defined on each slice of the three-dimensional data cube. The gross tumor volume (GTV) was defined as the macroscopic tumor visible on CT- and MRI-scans. A margin of 5 mm was added for the clinical target volume (CTV) and the planning target volume (PTV) was generated by adding 5 mm margin to the CTV. Margins could have been reduced in regions of directly adjacent radiosensitive organs at risk. Only two patients showed involved lymph nodes in recurrent situation. In one patient the lymph nodes were removed surgically prior to irradiation and therefore not included into the reirradiation volume, in the second patient the lymph nodes were included into the reirradiation volume and treated with the same dose as the local recurrence. No elective reirradiation of uninvolved regional lymph nodes was performed. Inverse treatment-planning was performed using the KonRad software developed at the German Cancer Research Center (DKFZ), which is connected to the 3D planning program VIRTUOS to calculate and visualize the 3D dose distribution. The IMRT treatment planning process has also been described in detail previously [17,20-23]. Reirradiation treatment was delivered by a Siemens accelerator (Primus, Siemens, Erlangen, Germany) with 6 or 15 MV photons using an integrated motorized multileaf collimator (MLC) for the step-and-shoot technique automatically delivering the sequences. The total doses were prescribed to the median of the PTV and usually the 95% isodose surrounded the PTV. An example of a three dimensional dose distribution is shown in figure 1. The prescribed dose ranged from 36 to 64 Gy with a median dose of 50.4 Gy in conventional fractionation (single dose 1.8-2 Gy, 5 fractions per week), resulting in a median cumulative dose of 112 Gy (range 91 - 134 Gy). Simultaneous systemic therapy was applied in 8 patients (platin-based chemotherapy in 7 patients, cetuximab in one patient). For detailed treatment characteristics see table 2. Doses to critical organs at risk were kept as low as possible. Assuming a 50% dose tolerance recovery of CNS structures from the initial treatment course, no patient received more than 60 Gy to the brainstem and 50 Gy to the spinal cord. For detailed information regarding the distribution of dose to organs at risk see table 3. Regular follow-up examinations took place in our institution or in the referring centers including at least clinical examination and CT or MRI of the head and neck region. Acute toxicity was scored according to CTCAE V3.0, late toxicity was scored according to RTOG criteria. In case of missing follow-up examinations, data was completed by calling the patient or the treating physician. Time to event data was calculated from the first day of radiation treatment until the last follow up information or until death. Response to treatment was based on CT or MRI findings 6 weeks to 3 months after the end of treatment according to RECIST criteria. Local control was defined as absence of tumor regrowth in the region of the treated recurrence on repeated CT or MRI scans based on best response after treatment. For example, if a patient had stable disease as best response after treatment, and no local progression occurred until the end of follow up or time of death, this patient was counted as locally controlled. Endoscopy findings or biopsy results were included into response assessment if available, but routine biopsies after treatment were not performed. In case of progression on imaging, endoscopy included biopsy if possible were performed. Locoregional control was defined as absence of tumor regrowth in the region of the treated recurrence and the bilateral cervical and supraclavicular nodal areas based on best response after treatment. In patients without further assessment of local or locoregional control e.g. after development of distant spread, the date of the last information about the local status was used for calculation. Local control (LC), Locoregional control (LRC), and Overall Survival (OS) were calculated using the Kaplan-Meier method. Differences in subgroups were tested for statistical significance by the log rank test. Differences were considered statistically significant for a p-value of ≤ 0.05.

**Table 1:** Patients characteristics

Patients characteristics	
**gender**	
male	14 (82%)
female	3 (18%)
	
**age (start of second RT course)**	
median	53
range	23-67
	
**KI (start of second RT course)**	
90-100%	10 (59%)
70-80%	6 (35%)
60%	1 (6%)
	
**primary T stage**	
Tx^a^	2 (12%)
T1	1 (6%)
T2	3 (18%)
T3	4 (24%)
T4	2 (12%)
unknown	5 (29%)
	
**primary N stage**	
N0	3 (18%)
N1	6 (35%)
N2	4 (24%)
N3	0 (0%)
unknown	4 (24%)
	
**histology (WHO)**	
I	0 (0%)
II	3 (18%)
III	14 (82%)
	
**recurrent T stage**	
rT1	3 (18%)
rT2	1 (6%)
rT3	1 (6%)
rT4	12 (71%)
	
**recurrent N stage**	
rN0	15 (88%)
rN1^b^	2 (12%)

^a^: 2 pts. were initially treated for cervical lymph nodes with cancer of unknown primary, in both the nasopharyngeal region had been included into the target volume, ^b^: one patient received neck dissection prior to reirradiation, in one patient the neck recurrence was included into the target volume of reirradiation, KI: Karnofsky index, age [years], staging according to UICC TNM classification 6^th ^edition

**Table 2:** Treatment characteristics

Treatment characteristics	
**primary RT course technique**	
2d	12 (71%)
3d	5 (29%)
	
**primary RT dose (boost)**	
median	66
range	50-72
	
**primary RT dose (nodal regions)**	
median	56
range	50-60
	
**second RT course technique**	
IMRT	14 (82%)
FSRT	3 (18%)
	
**second RT course dose**	
median	50,4
range	36-60
	
**second RT PTV volume**	
median	127
range	26-347
	
**time between RT courses**	
median	52
range	6-132
	
**cumulative dose both RT courses**	
median	112
range	91-132
	
**second RT course systemic therapy**	
concurrent chemotherapy	7 (41%)
concurrent immunotherapy	1 (6%)
adjuvant chemotherapy	2 (12%)
no chemotherapy	9 (53%)

all doses in Gy, IMRT: intensity modulated radiotherapy, FSRT: fractionated stereotactic radiotherapy, PTV volume in ccm, time between RT courses in months, chemotherapy platin-based in all patients, immunotherapy: cetuximab

**Table 3:** Maximum doses to critical organs at risk (OAR) during reirradiation for the entire cohort

Dose to OAR	median	min	max
brainstem	30	10	49
spinal cord	22	0	23
right optic nerv	25	1	43
left optic nerv	20	1	43
chiasma	11	1	39

all doses in Gy

## Results

The median follow up for the entire cohort was 20 months (6-84 months) and 31 months (10-84 months) in surviving patients.

### Response to Reirradiation

Local response was documented by CT or MRI scans in all patients. Complete response, defined as absence of measurable tumor, was found in seven patients (41%) and four patients showed partial response (24%) after treatment. Another five patients showed stable disease on repeated CT or MRI scans, whereas one patient had immediate progressive disease after 2 months and was counted as local recurrence. The group of patients with complete response included all patients with rT stage 1-3, all received reirradiation doses above 50 Gy, 5 of the 7 patients received concurrent chemotherapy and none of them developed a local recurrence so far. In contrast, all patients without complete response had rT4 stage, only 2 of them received irradiation doses above 50 Gy, concurrent systemic therapy had been administered in only 3 of them, and all local recurrences were found in that group.

### Local and Locoregional Control

**Figure 2:**
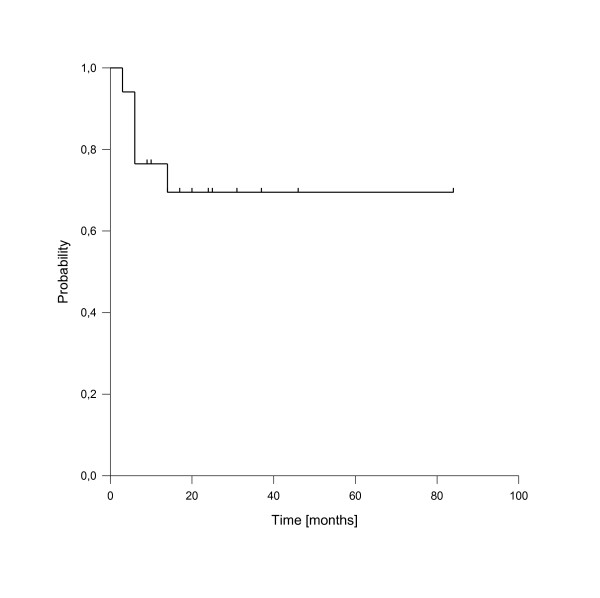
**Local control of the entire cohort**.

Five patients developed measurable isolated local recurrences after reirradiation, resulting in estimated 1- and 2-year local control rates for the entire cohort of 76% and 69%, respectively (see figure 2). Additional three patients suffered from isolated nodal recurrences in the neck region outside the reirradiation areas, resulting in combined 1- and 2-year locoregional control rates of 59% and 52%, respectively.

**Figure 3:**
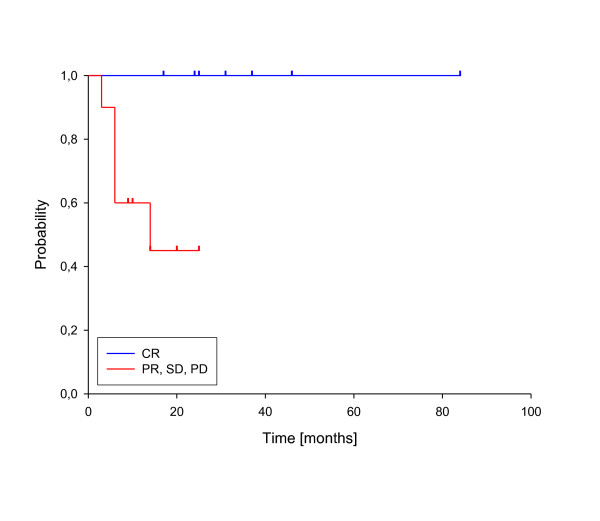
**Local control according to reirradiation response**. Complete Response vs. No Complete Response, p = 0.025.

**Figure 4:**
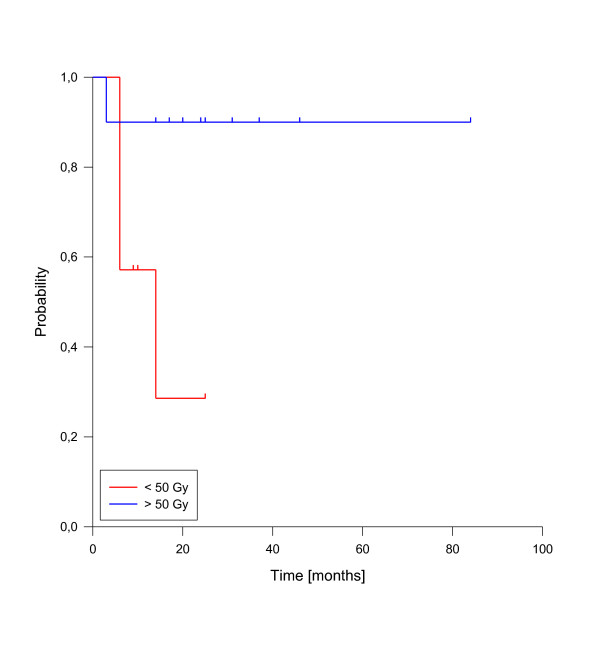
**Local control according to reirradiation dose**. Total dose < 50 Gy vs. > 50 Gy, p = 0.039.

**Figure 5:**
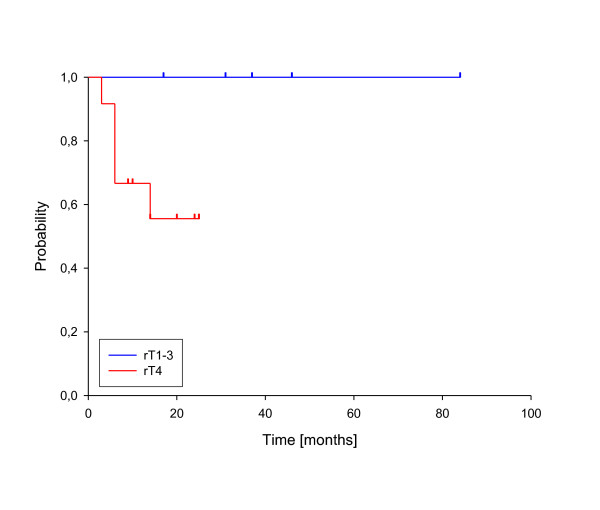
**Local control according to rT stage**. rT stage 1-3 vs. rT stage 4, p = 0.09.

**Figure 6:**
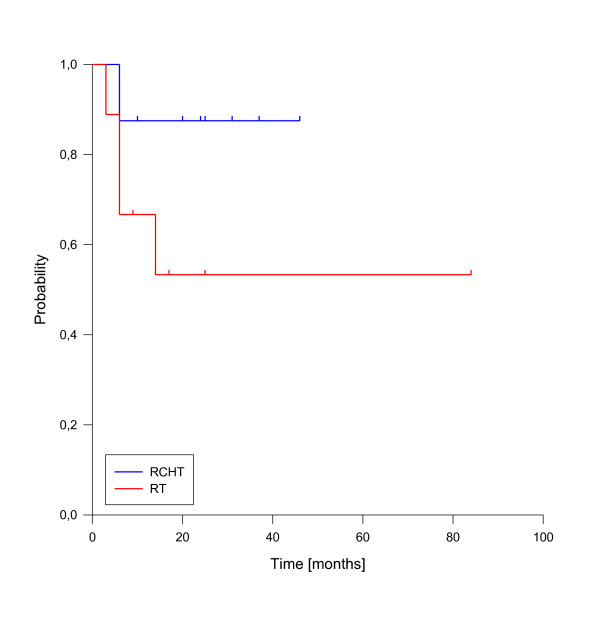
**Local control according to concurrent systemic therapy**. Concurrent systemic therapy vs. no radiotherapy alone, p = 0.16.

In univariate analysis, local control was significantly improved if complete response was achieved after reirradiation (p = 0.025, see figure 3). In fact, none of the patients with complete response has developed a local recurrence so far. A reirradiation dose of more than 50 Gy had also a significant impact on local control (p = 0.039, see figure 4). Trends to improved local control were seen for lower rT stage (rT1-3 vs. rT4, p = 0.09, see figure 5) and for concurrent systemic treatment (p = 0.16, see figure 6). Importantly, none of the patients with rT stage 1-3 developed a local recurrence so far.

### Overall survival and distant metastases

**Figure 7:**
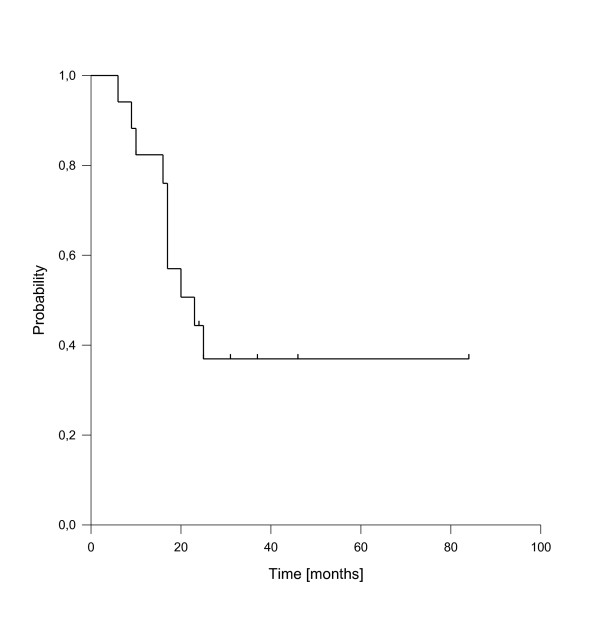
**Overall Survival of the entire cohort**.

**Figure 8:**
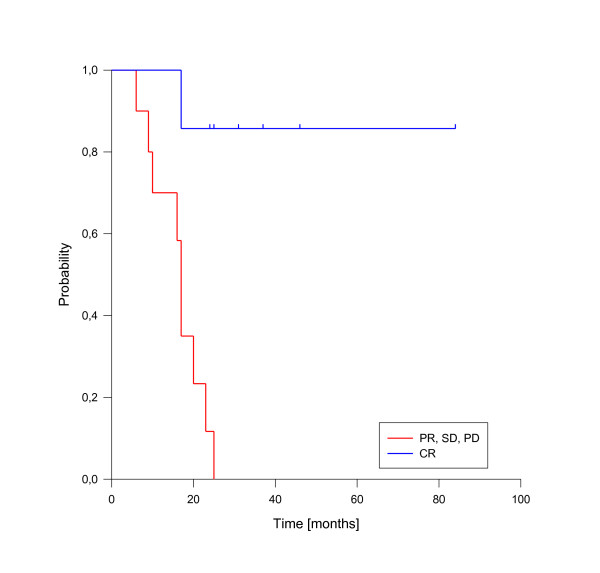
**Overall Survival according to reirradiation response**. Complete Response vs. No Complete Response, p < 0.001.

**Figure 9:**
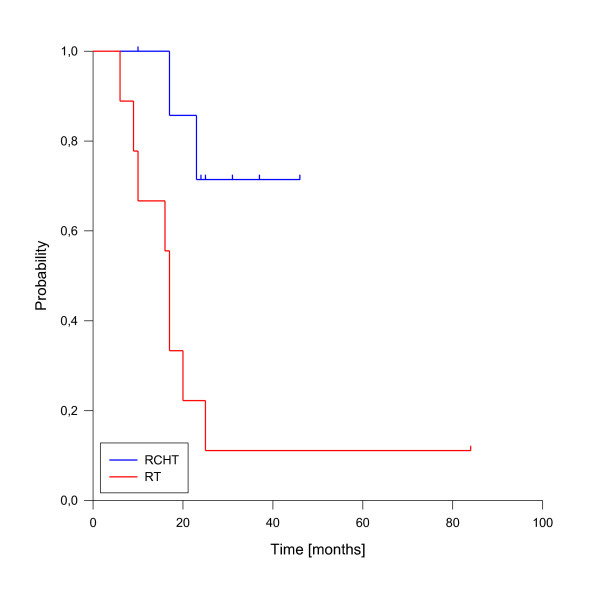
**Overall Survival according to concurrent systemic therapy**. Concurrent systemic therapy vs. no radiotherapy alone, p < 0.001.

**Figure 10:**
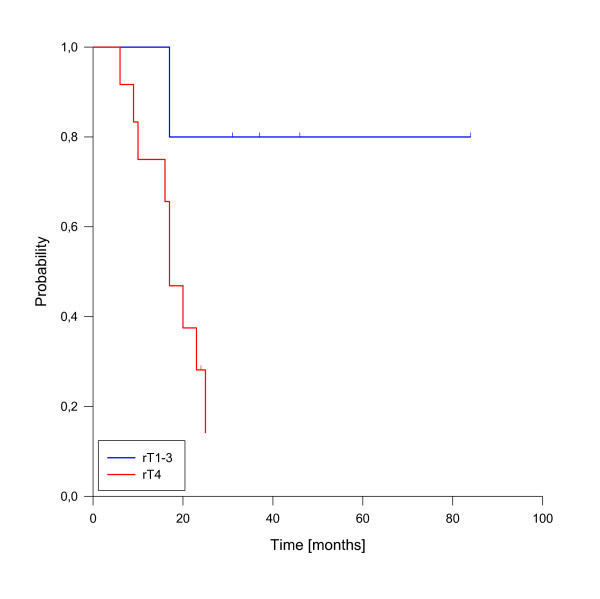
**Overall survival according to rT stage**. rT stage 1-3 vs. rT stage 4, p = 0.032.

**Figure 11:**
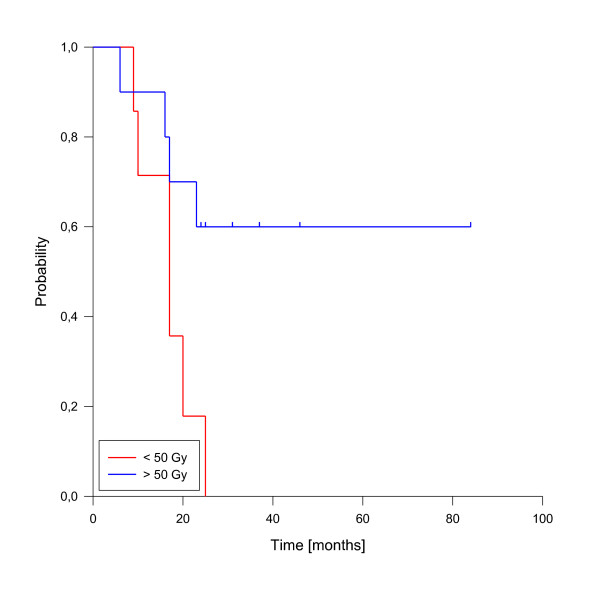
**Overall survival according to reirradiation dose**. Total dose < 50 Gy vs. > 50 Gy, p = 0.034.

A total of 10 deaths have been observed, all related to disease progression. The median estimated overall survival for the entire cohort was 23 months, transferring into 1-, 2- and 3-y-overall survival rates of 82%, 44% and 37% (figure 7). As for local control, the achievement of complete response was a strong prognostic factor for enhanced overall survival (p < 0.001, see figure 8). Only one patient of this group has died so far. Administration of concurrent systemic therapy also had a strong impact on overall survival (p < 0.001, see figure 9), with 6 of 8 patients in this group still alive. A low rT stage (p = 0.032, see figure 10) and a reirradiation dose above 50 Gy (p = 0.034, see figure 11) also significantly improved overall survival in univariate analyses. A total of six patients developed distant metastases during the follow-up period, three of them without locoregional recurrence. In one patient, distant metastases were confined to the lung, three patients suffered from liver metastases and in two patients multiple sites were involved at diagnosis of distant spread.

### Toxicity

Reirradiation was generally well tolerated with no or only minor acute toxicities mainly in patients with concurrent systemic treatment. Most patients developed a minor mucositis, a grade III mucositis was found only in two patients, with one of them suffering from a pre-existing perforation of the palate due to tumor infiltration prior to reirradiation. Mild radiation erythema (grade I) was found in 7 patients and one patient suffered from localized patchy moist skin desquamation (grade II). Three patients showed grade I/II leukopenia or thrombocytopenia, all had received concurrent chemotherapy. Nausea grade I/II was present in five patients, mainly attributed to chemotherapy also. One patients developed mild brain edema, which resolved after steroid medication. One patient suffered from a localized parodontal abcess formation and one from thrombosis of the axillary vein.

Late radiation toxicities from the first course of radiation treatment were relatively common and occurred in 65% of the patients, mainly as xerostomia or sensory alterations of taste, smell or hearing function. Late radiation toxicity attributable to the second course of radiation or worsening of pre-existing late toxicities were observed in the majority of patients but most of them were mild. Severe late radiation toxicities (grade III) attributable to reirradiation were found in 5 patients (29%). For detailed characteristics of late radiation toxicity see table 4.

**Table 4:** Severe late toxicities attributable to reirradiation

Late toxicity (grade III)	n
alteration of taste^a^	1
alteration of smell^a^	1
hearing loss^a^	2
cranial neuropathy	1
trismus	1
xerostomia^a^	1

^a^: late radiation toxicities in these categories which occured after the first course of radiation treatment and have not worsened after the second course were excluded, some patients developed more than one late toxicitiy

## Discussion

In the last decades combined chemoradiation has emerged as the standard of care in patients suffering from primary advanced nasopharyngeal cancer [1-6]. Local control and overall survival have further improved through the introduction of modern radiotherapy techniques which allowed dose escalation up to and beyond 70 Gy in conventional fractionation with an improved toxicity profile [24-28]. However, isolated local recurrence still remains an issue in about 10% of the patients and appears even more challenging to treat than in the past because of the intensified prior treatment in many patients. Currently available treatment options for recurrent nasopharyngeal cancer include surgery [29], chemotherapy [30] and various techniques of reirradiation like radiosurgery, fractionated stereotactic radiotherapy (FSRT), brachytherapy using mould or gold grain techniques and fractionated external beam radiotherapy [10-12,31]. Most of these options, namely surgery, radiosurgery and brachytherapy techniques yield excellent results in highly specialized, experienced centers, but are usually restricted to small volume recurrences confined to the nasopharynx and adjacent soft tissues [32]. In contrast, systemic treatment alone hardly results in long term survivors, and therefore it is usually restricted to patients with metastatic disease as a palliative treatment option [9,30]. For the remaining patient group, suffering from isolated but locally advanced recurrent lesions, external beam irradiation has been investigated using 2D or 3D treatment techniques, although this approach seems to be difficult due to the large numbers of important structures situated in the vicinity of a region that was already irradiated to a high dose during the primary treatment [32]. However, substantial local control rates have been reported by several groups, especially using modern 3D-RT techniques, but were commonly accompanied by high incidences of severe late toxicity [13-15]. For example, Zheng et al. [15] reported a 5-year-local control rate of 71% after treating recurrent nasopharyngeal cancer with 3D-RT up to 70 Gy, but grade 3 toxicitiy was found in all patients with half of them suffering from grade 4/5 side effects.

Intensity-modulated radiotherapy has been shown to yield superior dose distribution and sparing of organs at risk compared to 3D-conformal radiotherapy in many sites of the body. In the nasopharyngeal region, Hsiung et al. [16] also could show superior target coverage with less dose to organs at risk, especially brainstem and eyes, comparing IMRT with 5-field 3D-conformal RT used for boost or salvage irradiation. Excellent local control and overall survival rates beyond 90% have been reported by several groups using IMRT in the treatment of primary nasopharyngeal cancer with acceptable toxicity [24-28]. In our study, with the majority of patients suffering from locally advanced recurrences treated with moderate dose IMRT (median 50.4 Gy), we found a complete response rate of 41%, with 1-and 2-year local control rates of 76% and 69%, respectively. The corresponding overall survival rates decreased from 82% after one year to 44% after two years, probably due to the relatively high rate of distant metastases occurring during the follow-up period. It is possible that this patient group belongs to a subset of patients with primarily unfavourable biological tumor properties including the tendency for early metastasis. The incidence of severe late complications was 29%, which is lower than in most of the studies using 2D- or 3D-conformal radiotherapy [13-15], especially in terms of neurological side effects like temporal lobe necrosis, brain stem damage or cranial neuropathy. But despite the theoretically advantages of IMRT, only few clinical reports exist about the use of IMRT for recurrent nasopharyngeal cancer in the literature. For example Lu et al. [33] reported about 49 patients treated with high dose IMRT (68-70 Gy) which resulted in a 100% local control rate after a median follow up of 9 months. However, the incidence of late toxicities was not reported probably due to the very short follow-up time, excluding definite conclusions about the influence of late toxicity on the overall outcome. Chua et al. [7] reported on 31 cases treated with moderate dose IMRT (median dose 54 Gy) with very similar patient characteristics compared to our cohort considering age, gender, rT stage, time interval between the radiation courses and primary treatment. They found similar results, with a complete response rate of 58%, 1-year rates of locoregional control and survival of 56% and 63% respectively and a severe late toxicity rate of 19%, suggesting good short-term control with acceptable incidence of late side effects for this treatment concept. In a recent update, their initial results transferred into a 5-year local control rate of 27%-43% depending on rT stage, indicating reasonable long-term control and survival in a substantial proportion of patients in this unfavourable group with an acceptable toxicity profile [32].

Several prognostic factors have been discussed for outcome after reirradiation treatment of locally recurrent nasopharyngeal cancer, including age, performance score, histology, rT stage, tumor volume, time interval between radiotherapy courses, prior local failures, reirradiation dose and even EGFR-status [7,9,15,34-39]. As our study included only a small number of patients, conclusions considering prognostic factors should be drawn with caution, given the known limitations of univariate analyses in small cohorts. For example, it cannot be ruled out that differences in outcome according to treatment factors, for example radiation dose or simultaneous application of systemic therapy were biased by a tendency to intensified treatment in less advanced lesions. However, despite the small number of patients in our study we found an impact of rT stage, tumor response, reirradiation dose > 50 Gy and simultaneous use of chemotherapy for local control and/or overall survival, whereas time interval between the RT courses, age, gender and performance score showed no prognostic value in our analysis.

The most consistent prognostic factor being reported is rT stage [7,9,15,35,36]. Especially patients with invasion of intracranial structures (rT4) had a particularly poor local control and overall survival in most of the series. In our cohort, all local recurrences were seen in patients with rT4 stage resulting in 1- and 2-year local control rates of only 67% and 56% and a 2-year overall survival of only 28%. Chua et al [7] reported an even more distinguished difference in their cohort with a 1-y-locoregional control rate of only 35% in patients with rT4 stage compared to 100% in rT1-3 stage patients, but in contrast to our study, rT stage had no prognostic value for overall survival in their series. The poor outcome of patients with rT4 stage with respect to local control and overall survival is possibly related to different factors. The decreased local control may be caused by suboptimal target coverage due to the constraints of the nearby critical structures and the tendency to lower overall dose prescription in heavily pre-treated patients with advanced lesions in the fear of excess toxicity. However, although the difference in overall survival could obviously be at least partly attributed to uncontrolled local disease, these patients could also have a higher risk of regional and distant failure per se. In our study only one out of five patients with rT1-3 stage, but 5 out of 12 patients with rT4 stage developed distant metastasis after reirradiation.

The second consistently reported prognostic factor is reirradiation dose [9,15,35,38,39]. Several authors described improvements in outcome for reirradiation doses above 60 Gy [9,15,35], but in some of these series high local control did not transfer into improved survival and was rather accompanied by high rates of severe complications probably responsible at least in part for this difference. For example Zheng et al. [15] achieved an excellent 5-year local control rate of 71% in a cohort of less advanced lesions, whereas the 5-year overall survival rate was only 40%. Eleven of their 86 patients died without signs of disease progression but showed severe complications. In contrast, Chang et al. [39] observed that a dose > 50 Gy already yielded better survival in their series. In our cohort, doses above 50 Gy were associated with improved local control and overall survival, but this result could be influenced by a tendency to apply higher doses in patients with less advanced rT stage. However, for patients suffering from rT1-3 tumors, reirradiation doses of 50-60 Gy resulted in excellent short term local control and overall survival in our and other reported series [7], while in rT4 stage patients doses of about 50 Gy seem to have a palliative value only in most cases. However, whether further dose escalation in those advanced patients will improve outcome remains uncertain. According to our experience, sparing of adjacent organs at risk can be difficult in rT4 patients even with the use of IMRT and further dose escalation would probably distinctly increase late toxicity. One way to improve outcome could be the use of newer radiotherapy techniques like protons or heavy ions, which could allow a superior dose distribution and sparing of normal tissues. For example, Taheri-Kadhoda et al. [40] showed a superior dose distribution in the nasopharyngeal area with respect to target coverage and dose to organs at risk with 3-field intensity modulated proton therapy compared to 9-field step and shoot photon IMRT.

Another possible way to improve outcome could be combined modality treatment using induction and/or concurrent systemic therapy. In our series, we found a trend to improved local control and a significant improved overall survival in patients receiving concurrent systemic treatment. Another series reported by Chua et al. [41] showed a 1-year local control rate of 75% in advanced recurrences after induction chemotherapy with cisplatin/gemcitabine followed by reirradiation with IMRT. While induction chemotherapy could not only allow better target coverage and sparing of adjacent organs at risk through tumor shrinkage, it may also be used to delay the second course of RT in patients who relapse shortly after primary treatment [32]. Concurrent chemotherapy has been shown to improve response, local control and overall survival compared to radiotherapy alone in the treatment of primary nasopharyngeal cancer in many series and is now accepted as the standard of care for advanced primary lesions [1-6]. In recurrent nasopharyngeal cancer it could also improve the rate of complete responses, which had a significant impact on local control and overall survival in our series. Patients with complete response showed a 2-year local control rate of 100% and a 2-year OS rate of 86% compared to 45% and 12% in the group with residual disease. Therefore combined modality approaches including induction and/or concurrent systemic treatment could not only be used to further improve outcome especially in advanced (rT4) recurrent lesions but also for patient selection processes.

In conclusion, reirradiation using IMRT for local recurrences of nasopharyngeal cancer with moderate doses yields excellent results in terms of local control and overall survival in rT1-3 lesions. Acute and late toxicity seems to be reduced compared to the results published with 2D- or 3D conformal treatment approaches. However, outcome in locally advanced recurrent lesions (rT4) is still limited and treatment with doses in the range of 50 Gy has to be considered palliative in the majority of cases. Therefore close monitoring of patients after primary treatment of nasopharyngeal cancer should be mandatory in order to detect local recurrences early enough to offer salvage options with curative intent. Combined modality approaches or newer radiation technologies like protons or heavy ions should be further investigated especially considering advanced recurrent lesions.

## Conflict of interest Notification

The authors declare that they have no competing interests.

## Authors' contributions

FR participated in data acquisition, literature review and drafted the manuscript. FZ, LSE, CTI and CTH participated in data acquisition and literature review. MB, JD and PEH participated in drafting the manuscript and revised it critically. All authors read and approved the final manuscript.
